# An Incompatible Mixture

**Published:** 1899-05

**Authors:** 


AN INCOMPATIBLE MIXTURE. Iodide of potassium added to a solution of arsenite of potash forms an incompatible mixture, the arsenic being precipitated in the form of an oxyiodide of arsenic.

